# Coupling Droplet Microfluidics with Mass Spectrometry for Ultrahigh-Throughput Analysis of Complex Mixtures up to and above 30 Hz

**DOI:** 10.1021/acs.analchem.0c02632

**Published:** 2020-07-30

**Authors:** Emily E. Kempa, Clive A. Smith, Xin Li, Bruno Bellina, Keith Richardson, Steven Pringle, James L. Galman, Nicholas J. Turner, Perdita E. Barran

**Affiliations:** † Michael Barber Centre for Collaborative Mass Spectrometry, Manchester Institute of Biotechnology, Manchester M1 7DN, United Kingdom; ‡ Sphere Fluidics Limited, McClintock Building, Suite 7, Granta Park, Great Abington, Cambridge CB21 6GP, United Kingdom; § Waters Corporation, Stamford Avenue, Altrincham Road, Wilmslow SK9 4AX, United Kingdom; ∥ Manchester Institute of Biotechnology, Manchester M1 7DN, United Kingdom

## Abstract

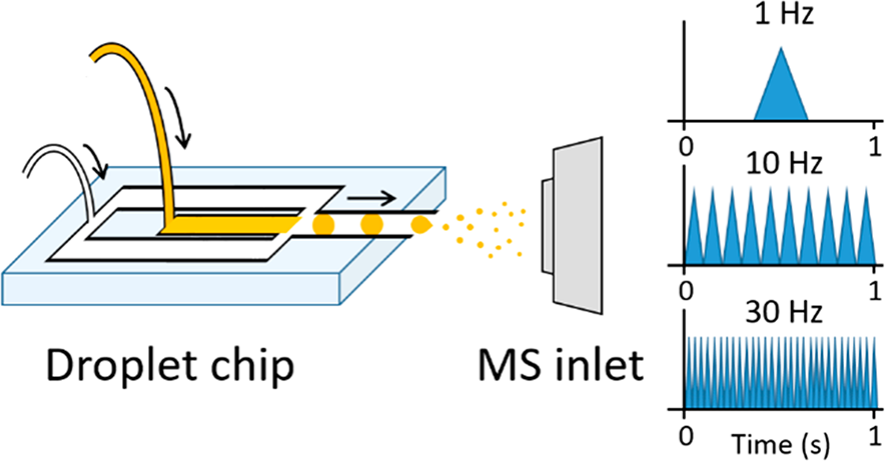

High- and ultrahigh-throughput label-free sample analysis is required by many applications, extending from environmental monitoring to drug discovery and industrial biotechnology. HTS methods predominantly are based on a targeted workflow, which can limit their scope. Mass spectrometry readily provides chemical identity and abundance for complex mixtures, and here, we use microdroplet generation microfluidics to supply picoliter aliquots for analysis at rates up to and including 33 Hz. This is demonstrated for small molecules, peptides, and proteins up to 66 kDa on three commercially available mass spectrometers from salty solutions to mimic cellular environments. Designs for chip-based interfaces that permit this coupling are presented, and the merits and challenges of these interfaces are discussed. On an Orbitrap platform droplet infusion rates of 6 Hz are used for analysis of cytochrome *c*, on a DTIMS Q-TOF similar rates were obtained, and on a TWIMS Q-TOF utilizing IM-MS software rates up to 33 Hz are demonstrated. The potential of this approach is demonstrated with proof of concept experiments on crude mixtures including egg white, unpurified recombinant protein, and a biotransformation supernatant.

High-throughput screening (HTS) and ultrahigh-throughput screening (uHTS) methodologies aim to analyze tens to hundreds of thousands of samples per day.^1−5^ In both industry and academia, the use of microtiter plate formats has become ubiquitous for sample handling and HTS. This format is used across many different analytical platforms such as fluorescent readers^6^ and liquid chromatography injection systems.^7,8^ Label-free detection strategies are often coupled to microtiter plates via robotics and measure the intrinsic physical properties of the sample in contrast to those based upon ligated chromophores (fluorescent or color metric) or radioisotopes.^9,10^ Workflows which fulfill both “label-free” and high-throughput prerequisites are highly sought after by coupling the highest throughput analytical instrumentation currently available with robotics.

In recent years developments in microfluidics have shown that it is possible to reproducibly manipulate volumes of liquids within channels measuring less than 1 mm in diameter.^11−13^ Droplet microfluidics, in particular, involves the transport and study of compartmentalized “bursts” of analyte formed by the transport of two immiscible phases with droplet generation often occurring “on-chip”.^14−18^ Microfluidic devices, or chips, are often fabricated from glass, polymers, or silicon^15,19^ with inbuilt channels that facilitate the movement of droplets through the device toward the analytical technique in operation. Previously, droplet microfluidic chips have been successfully coupled to a wide range of analytical instrumentation, including fluorescence^20^ and optical detection,^21^ mass spectrometry,^22,23^ Raman spectroscopy,^24^ and NMR,^25^ with each droplet considered as an individual sample or reaction vessel. Combining these techniques with microfluidics supplied analyte at speeds up to 10 000 droplets/s^26^ and facilitates high-throughput screening in an alternative arrangement to microtiter plate formats.

Mass spectrometry (MS) is a highly sensitive, “label-free” analytical technique widely employed to qualitatively and quantitatively probe the composition of a sample. Acquisition speed is analyzer dependent, and this is determined by the physics, electronics, software and manufacturer, to some extent the operator, the mass and charge upon ions in question, and the mass resolution required. The coupling of automated sample introduction with mass spectrometry is not new,^27,28^ although as higher throughput analysis is required analyzers and acquisition modes have become faster and faster. Time of flight (TOF) mass spectrometers inherently have the highest acquisition speeds without compromising resolution^29^ and are most obviously suited to HTS applications.

Coupling of HTS microfluidics to mass spectrometers is commonly achieved through the incorporation of a liquid outlet similar to that of an electrospray (ESI) or nanoelectrospray (nESI) emitter into a chip, allowing for direct infusion of the analytes into the ion source.^30−32^ Droplet microfluidics directly coupled with MS has been hindered by the need to extract or divert the analyte-containing phase (commonly aqueous) from the separative phase (commonly hydrophobic) prior to MS infusion.^33−35^ Separative phases can contaminate MS instrumentation, and dual-phase fluidics can lead to Taylor cone instability and inadequate electrospray ionization. A number of reports in which a dual-phase system has exploited the alternating aqueous and oil phases exiting the microfluidic device for droplet detection have been highlighted. Smith et al.,^23^ Wink et al.,^22^ and Steyer et al.^36^ all directly infuse both oil and aqueous streams directly into the MS instrumentation through varying emitter types and display the MS detection of individual droplets. High-throughput microdroplet infusion with MS detection for HTS with a throughput of up to 10 Hz was reported by Steyer et al. in 2019; we note that this was implemented in selected ion monitoring mode,^36^ with commensurate sensitivity gains, compared with measuring a full mass spectrum.

The majority of literature entries only report the adaption of microdroplet microfluidics with one ESI MS platform; however, here we illustrate flexibility through chip–MS coupling to instruments from three different vendors. We demonstrate how MS droplet screening can be extended to rates over 30 microdroplets/s using fast scanning acquisition IM-Q-TOF instrumentation. We envision such a platform could be utilized in biotechnology to detect reaction products along with the modified enzyme. This would have particular relevance to directed evolution studies if mass spectrometry could directly inform on the nature of successful mutation(s) in the evolved enzyme and prevent a subsequent PCR step.

## Methods and Materials

All standards (l-tyrosine, leucine enkephalin, bovine ubiquitin, equine cytochrome *c*, and bovine serum albumin (BSA)) were purchased along with ammonium acetate from Sigma-Aldrich (Dorset, UK). Leucine enkephalin was dissolved in deionized water (obtained from a Milli-Q Advantage ultrapure water filtration system, Merck Millipore, Darmstadt, Germany) containing 0.1% formic acid (Fisher Scientific, Loughborough, UK) to produce a ∼1.3 mM solution of the peptide. Other standard materials (proteins and small molecules) were dissolved in a solution of 100 mM ammonium acetate in deionized water to produce ∼100 μM solutions of each standard (unless stated otherwise). Preparation of an egg white solution required separation of the egg white from the yolk prior to dilution in 1 M ammonium acetate solution (1:5 v/v) before vortexing for ∼30 s.^37^ Whole cell biotransformations were performed upon addition of a substituted cinnamic acid species (5 mM) to the phenylalanine ammonium lysate (PAL) cell paste suspended within a 4 M solution of ammonium carbonate and incubated at 30 °C, 250 rpm for 24 h. For analysis, the resulting solution was centrifuged (5 min, 13 000 rpm) to remove insoluble cellular material and the supernatant diluted to 800 mM ammonium carbonate with 100 mM ammonium acetate solution. In every case, the separative oil phase consisted of Pico-Surf 1 (Sphere Fluidics Ltd., Cambridge, UK) diluted to 1% in Novec 7500 Engineered Fluid (3M, Maplewood, MN, USA).

### Chip Design and Fabrication

All microfluidic chips used in this work were fabricated from polydimethylsiloxane (PDMS, Dow Chemical Co., MI, USA) using established photolithography and soft lithography techniques as described in the literature.^13,38^ A detailed procedure can be found in the Supporting Information. Stainless-steel capillaries (Vita Needle Co., Needham, MA, USA) of varying lengths with an internal diameter of 76 μm were incorporated into the fluidic outlet channel of the final PDMS devices and secured using Elastosil E43 silicon sealant (Wacker Chemie AG, München, Germany) as described by Wink et al.^22^

### Coupling to ESI Sources and Establishing Droplet Flow

**Figure 1:**
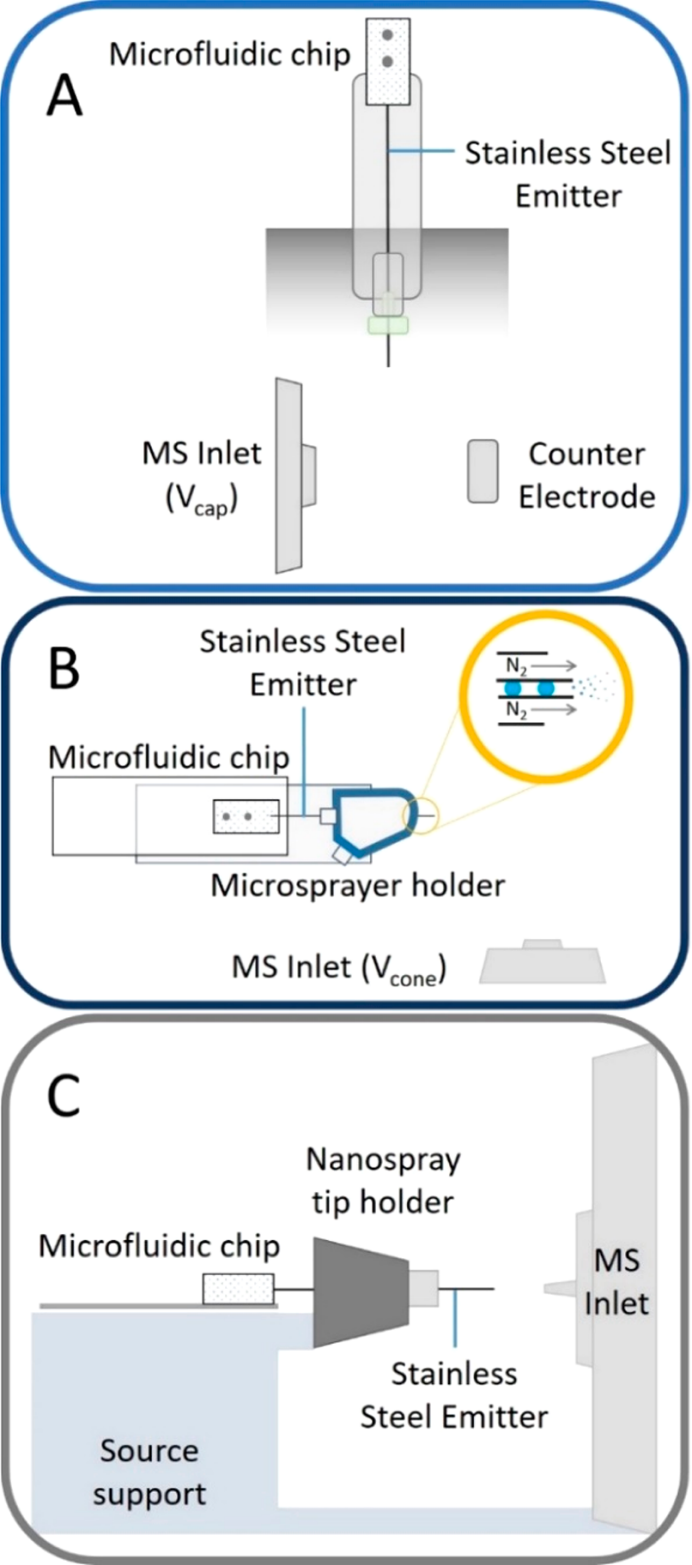
(A) Schematic (side view) of the adaptation of a vertically mounted Agilent Nanospray ESI source to incorporate a microfluidic chip. Emitter is grounded and held ∼0.3 cm from a counter electrode held at ∼1.75 kV. Entire assembly is enclosed from the lab. (B) Schematic representation (top view) of the microfluidic chip interfaced to a Waters z-spray source by adapting a microspray assembly (source support not shown). Close up (yellow ringed inset) indicates coaxial gas flow around the stainless-steel emitter. Emitter is held at ∼2.8 kV and positioned 0.5 cm from the conical counter electrode which is the entrance to the mass spectrometer held at *V*_cone_ (∼54 V). (C) Schematic (side view) of the droplet microfluidic chip interfaced with the Thermo Fisher Q Exactive nESI source in which the stainless-steel emitter is inserted in the place of the nanospray tip and held in place with a conductive screw. Distance between the emitter and the entrance to the MS is 0.5 cm. These schematics are not to scale. Photographic representations indicating the scale and dimensions of the microfluidic chip within all 3 instrumental configurations can be found in the Supporting Information Figures S3, S4, and S6.

Infusion to each mass spectrometer was achieved through the coupling of a designed chip to the respective vendor’s nESI source (Figure 1). Exact coupling methods differ as described, and all experiments were undertaken in positive ionization mode. The oil and aqueous connections required to generate droplets with the microfluidic chip consisted of 1.09 mm o.d. tubing (0.38 mm i.d., Smiths Medical Inc., Minneapolis, MI, USA) between the punched chip inlets and the syringe pump (neMESYS low-pressure syringe pump, CETONI GmbH, Korbußen, Germany) in each case. As droplets are generated with a diameter larger than that of the internal diameter of the stainless-steel emitter, droplets and the segmented oil phase reach the outlet of the emitter as “plugs” of that phase and as such do not lose their interdroplet spacing as they enter the mass spectrometer. Prior to infusion into the mass spectrometer, the frequency of the generated droplets and their diameter were determined via optical analysis. This was achieved through the use of the Picodroplet Single Cell Encapsulation System instrumentation (Sphere Fluidics Ltd., Cambridge, UK). Observed frequencies are dependent on the device dimensions and the flow rates utilized during infusion, and consistency of droplet frequency for a given flow rate can be used to validate the manufacturing process. Chip designs of varying channel dimensions were used in this study to generate droplets at differing frequencies and dimensions. Note that the chips used are interchangeable between instruments with the design chosen in each case due to device availability only.

Infusion of the oil and aqueous phases allowed droplet generation with the droplet emulsion exiting the outlet of the stainless-steel emitter able to be observed by the naked eye. Upon application of the electrospray voltage, fluid reaching the outlet of the emitter can be seen to enter the MS inlet in the form of an electrospray plume (see Figure S3F for an example of this). As microdroplets enter the mass spectrometer individually, increases in the mass spectrometry signal are observed in the total ion and extracted ion chromatograms. If the instrumental acquisition speed is sufficient, each droplet is observed as a peak in the chromatogram, with peaks arising at the rate of droplet generation.

### DTIMS Q-TOF Coupling

Interfacing the droplet microfluidic chip with an Agilent 6560 IM-Q-TOF (Agilent Technologies, Santa Clara, CA, USA) required incorporation of a stainless-steel emitter of approximately 12 cm in length into the device. The chip was carefully removed from a supporting glass slide and the stainless-steel emitter threaded through a metal union, conductive ferrule, and finger tight screw before being placed in the nESI source probe as indicated in the photograph in Figure S3B. The outer casing of the nESI probe was replaced, and the probe was inserted vertically into the source (Figure 1A). The position of the stainless-steel capillary emitter between the MS inlet and the counter electrode can be observed via the internal camera.

**Figure 2:**
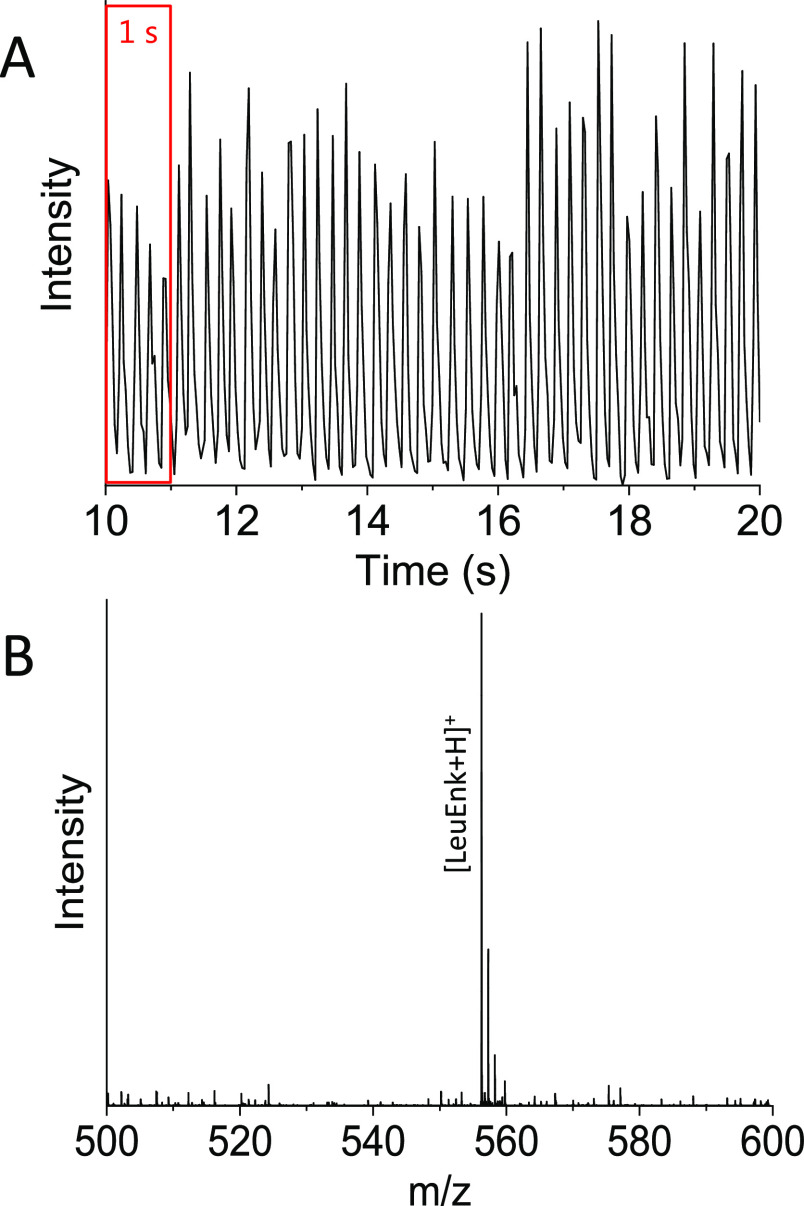
Total ion chromatogram (TIC) acquired during infusion of droplets (∼2.1 nL) containing leucine enkephalin (LeuEnk, ∼1.3 mM solution) at an infusion rate of approximately 5 droplets/s (Hz). Each individual peak indicates one droplet reaching the Agilent 6560 IM-Q-TOF detector. Mass spectrum (*m*/*z* range 500–600) acquired from one droplet containing LeuEnk ([LeuEnk + H]^+^ = 556.27 Da).

Figure 2 shows droplet infusion from a solution of leucine enkephalin occurring at ∼5 Hz (optical analysis data not shown) with a commensurate frequency for mass spectrometry detection as determined by the total ion chromatogram (TIC Figure 2A). Akin to chromatography, a mass spectrum can then be extracted for an individual droplet. Figure 2B shows the mass spectrum of leucine enkephalin acquired from a single droplet. The Agilent Q-TOF acquisition range is restricted to ±50 mass units from the parent ion mass of intact leucine enkephalin (*m*/*z* 556.27) to facilitate enhanced sensitivity for targeted detection of the species of interest. To detect each droplet produced at 5 Hz, the Q-TOF scan speed was set to 35 scans/s in the acquisition software giving ∼7 scans per droplet TIC. This scan rate is sufficient to delineate the analyte signal from each droplet, and the maximum permitted scan rate (50 scans/s) provides a little more resolution between droplets. For higher droplet infusion frequencies (10 Hz and above), the resolution is compromised and a faster acquisition system would be needed to capture all of the mass spectrometry information from each droplet.

### TWIMS Q-TOF Coupling

The microfluidic chip was mounted on a Waters nESI source with a microsprayer for infusion into a Waters SYNAPT G2Si Q-TOF (Waters Corp., Milford, MA, USA) (Figure 1B). Due to the dimensions of the microsprayer device, a shorter stainless-steel emitter (approximately 6 cm) was incorporated into the droplet generation device, which also reduces the back pressure on the droplets. Upon insertion of the stainless-steel emitter to the microsprayer assembly, the emitter was fastened in place by tightening the supporting screw and the glass slide secured to the base of the microsprayer using Blu Tack (Figure 1B). A ∼1 mm protrusion of the stainless-steel emitter from the microsprayer outlet was found to be optimal for stable electrospray.

Mounting of the microsprayer–chip construct onto the Waters nESI source XYZ stage (Figures 1B and S4B) allowed the emitter to be optimally positioned perpendicular to the source inlet cone. As droplets are generated and reach the end of the stainless-steel emitter, the electrospray voltage (∼2.8 kV) applied directly to the emitter allows for the generation of an electrospray plume. This is assisted by a coaxial flow of nitrogen (1.5 bar) (Figure 1B, insert). As for data obtained from the Agilent 6560 IM-Q-TOF instrument (Figure 2A), droplet peaks in the TIC are observed at a frequency close to that of droplet generation. A TIC obtained from this instrument is indicated in Figure S5B, with droplet generation occurring at a rate of approximately 9 Hz. The acquisition speed utilized during this experiment was equal to 0.016 s with an interscan delay of 0.010 s, corresponding to ∼38 scans/s. This is the maximum permitted speed for MS data acquisition on this platform. Figure S5C shows the extracted mass spectrum obtained from 1 of these droplet TICs, indicating that under these conditions, as for nESI from an equivalent concentration of aqueous ammonium acetate, the major charge ions observed for this protein are [M + 6H]^6+^ and [M + 5H]^5+^ (Ubiquitin intact mass ∼8.6 kDa).

### Sensitivity Analysis Using TWIMS Q-TOF

Sample concentrations of the solutions analyzed in Figures 2, S5, and S7 are all in excess of 100 μM. When expressing detection limits for such a dual-phase system, not only must the solution concentration be considered but also the droplet size must be too. For example, the droplets infused at 9 Hz during the experiment described above had approximate volumes of 0.8 nL and a ubiquitin concentration of 100 μM (Figure S5), which equates to detection of ∼700 pg of protein per droplet. Lowering the concentration to 5 μM corresponds to ∼150 pg of protein per droplet (Figure S8), albeit the lower infusion rates and slightly differing chip dimensions give droplets 3.6 nL in volume. We envisage that the detection limits for solutions below 5 μM are possible with both MS and microfluidic chip optimization but caution that absolute limits will be droplet size, instrument, and analyte specific.

### Orbitrap Coupling

Interfacing the microfluidic chip with the Thermo Fisher Scientific Q Exactive (Waltham, MA, USA) nESI source followed a similar approach to that for the Waters instrument above. The chip, mounted upon a glass slide, incorporated a ∼6 cm stainless-steel emitter, which was inserted through the rear of the nanosource tip holder and secured in place using a stainless-steel nut (Figure 1C). The emitter position can be adjusted using the *XYZ* stage. In this arrangement, the electrospray voltage (∼2.4 kV) is applied continuously to the chip emitter as droplets are being generated and subsequently infused.

A similar result to that of the previous instruments discussed is observed (e.g., Figure 2A) with microdroplets appearing as discrete peaks in the EIC as they are infused (an example EIC from this instrumentation can be found in Figure S7B). The irregularity in droplet frequency and intensity is attributed to a mismatch between the acquisition frequency and the droplet infusion rate, whereby the acquisition of data (comprising both AGC and trap fill time) occurs at intervals which do not exactly coincide with the presence of a droplet. In order to obtain the maximum scan rate of this instrument, a decrease in the instrument resolution is required, whereupon an instrument scan rate of 30.3 Hz is attainable. For microdroplet infusion in the range of 6 Hz (as seen in Figure S7B) such an acquisition rate is achievable; however, the lack of resolving power means that for massive ions the isotopic resolution is lost. This is demonstrated here for the ∼12.2 kDa protein cytochrome *c* (inset, Figure S7C). This is a feature of FT-MS, and if isotopic resolution is required, coupling Orbitrap instruments in their current inceptions to such high-throughput sample delivery will be limited to small molecule and more targeted detection.

### Expansion of Sample Scope

**Figure 3:**
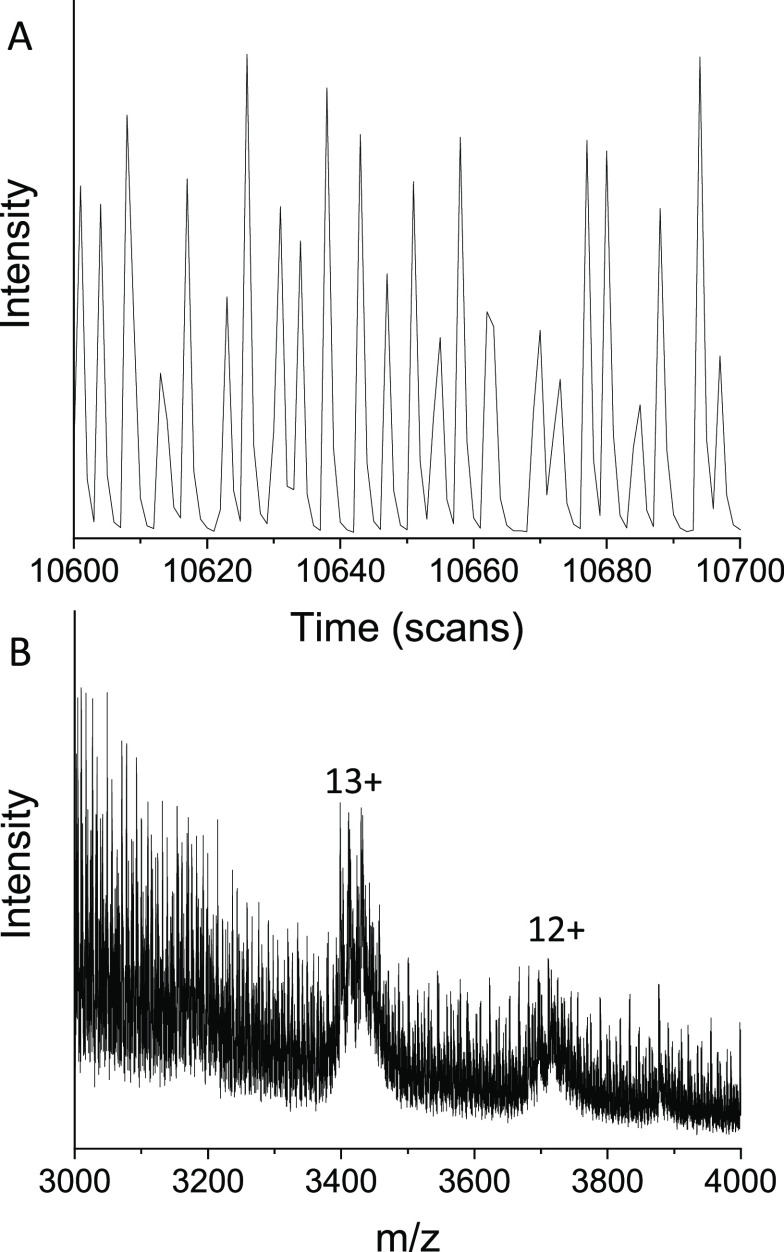
Data for the infusion of droplets containing egg white in aqueous ammonium acetate solution (1 M) obtained using TWIMS Q-TOF instrumentation. (A) Total ion chromatogram of infused egg white droplets; 100 scans equivalent to ∼2.6 s are shown (MS total cycle time = 0.026 s/scan). (B) Mass spectrum (unmodified) obtained for the infusion of egg white droplets upon combining ∼8 min of acquisition. Ovalbumin protein (44 kDa) from egg white has been identified in the spectrum with the major charge states of ovalbumin monomer (12+ and 13+) indicated.

Our goal is to infuse microdroplets that contain crude reaction mixtures and use HTS to monitor biocatalytic processes both at the product and at the modified enzyme level. To work toward this we chose to examine dilute egg white (Figure 3). As with purified samples, a total ion chromatogram is obtained with each peak arising corresponding to the infusion of 1 droplet (Figure 3A). Ovalbumin, a major protein (∼44 kDa) found within egg white, is clearly present in the corresponding mass spectra (Figure 3B), which also has the form of a natively folded protein, possessing a narrow charge state distribution. A similar TIC is observed when infusing a crude lysate of a recombinant nanobody (Figure S11) and also the 66 kDa protein BSA (bovine serum albumin, Figure S10) infused from a native MS solution.

**Figure 4:**
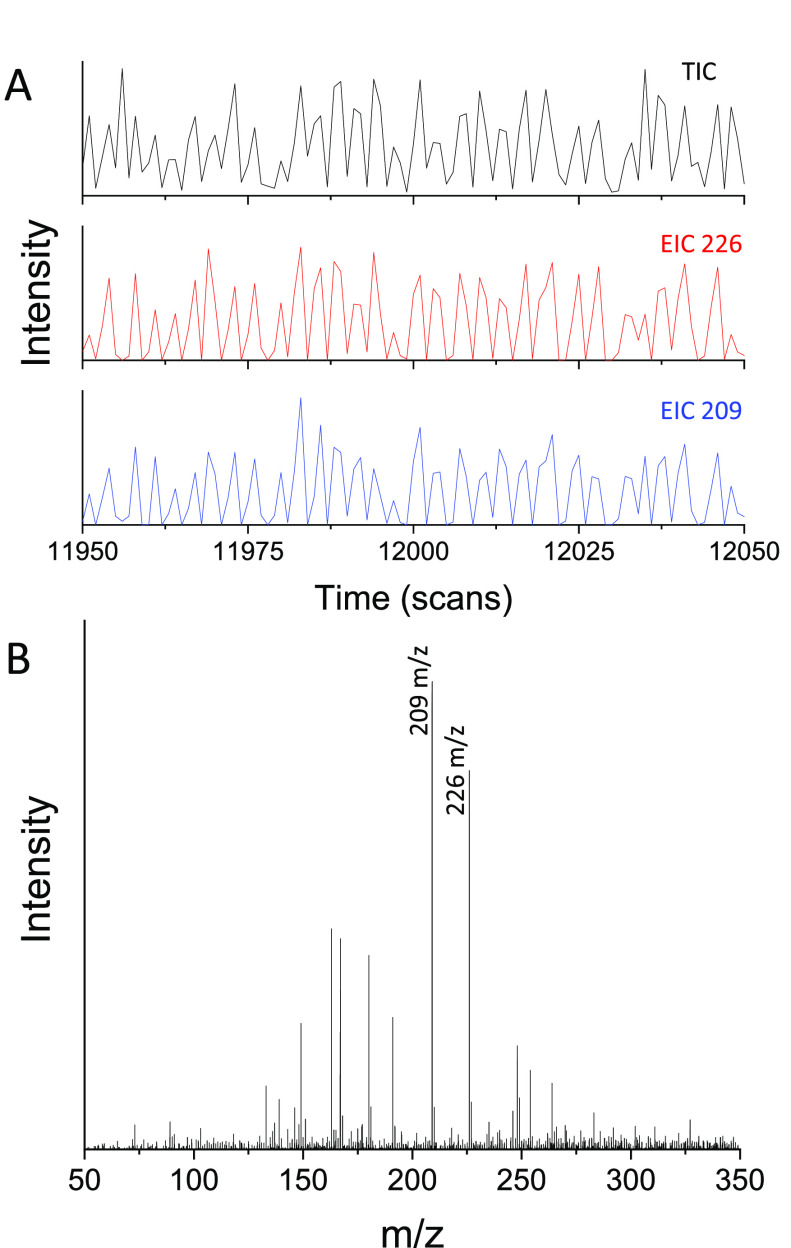
Data for the infusion of droplets containing phenylalanine ammonium lyase (PAL) biotransformation supernatant in aqueous ammonium acetate solution (100 mM) obtained using TWIMS Q-TOF instrumentation. (A) Total and extracted ion chromatograms obtained from infused supernatant droplets. One hundred scans equivalent to ∼2.6 s are shown (MS total cycle time = 0.026 s/scan). (B) Mass spectrum obtained from 1 supernatant droplet indicating detection of the biotransformation starting material (*m*/*z* 209) and product (*m*/*z* 226).

Detection of small molecules within a biotransformation supernatant at 800 mM ammonium carbonate is demonstrated in Figure 4. A TIC trace and EIC traces for both the reaction starting material and the product (Figure 4A) are obtained following infusion of the reaction mixture with 1 droplet MS data obtainable (Figure 4B). It is noted that the TIC traces obtained for these high-salt solutions (Figures 3 and 4) can differ from those for standard solutions (Figures 2, S5 S7, and S9) by way of their peak-to-peak (i.e., droplet-to-droplet) repeatability. The crude mixtures show more variation in droplet peak area, and the frequency of the incoming droplets is not as consistent as its standard solution counterparts. We anticipate these differences are attributable to the higher viscosities of these solutions, thus altering the generation frequency of droplets within the chip at the flow-focusing junction. In addition, the increased salt concentrations are a likely cause of electrospray instabilities at the emitter outlet in droplet mode (although this is not seen in direct infusion). Despite this, full mass spectra are obtained from single droplets, and the broad scope of such assignments demonstrates the platform’s label-free capabilities.

All experimental work described here exploits detection of a full *m*/*z* scan range as opposed to a selected ion approach taken by Steyer et al.^36^ Utilization of a full scan also prevails over alternative detection methodologies such as fluorescence due to its ability to detect and distinguish multiple analytes simultaneously. Selected ion mode of course has a role to play, and here, we have shown that we can *m*/*z* select individual charge states of protein ions, which would be the first step toward a top-down sequencing strategy to identify mutations in a given enzyme (Figure S13).

### Fast Scanning Acquisition–TWIMS Q-TOF

To increase the throughput achieved upon the Waters SYNAPT G2Si instrument, a faster scanning acquisition mode was implemented as a variant of the SONAR acquisition mode developed by Waters for rapid data-independent acquisition.^39^ In this mode, the instrument is essentially operating in a standard MS mode, but additional spectra are accumulated using the SYNAPT’s ion mobility acquisition architecture. In this way, one acquisition cycle comprises 200 sequentially acquired “spectral bins” obtained in the same time as one original MS scan. This allows a potential increase from ∼38.5 to 7700 spectra/s; however, for the purpose of these “proof of concept” experiments, the acquisition cycle time was fixed at 1 s. Therefore the acquisition rate was equivalent to 200 spectra/s, representing an approximately 5-fold increase in sampling points.

**Figure 5:**
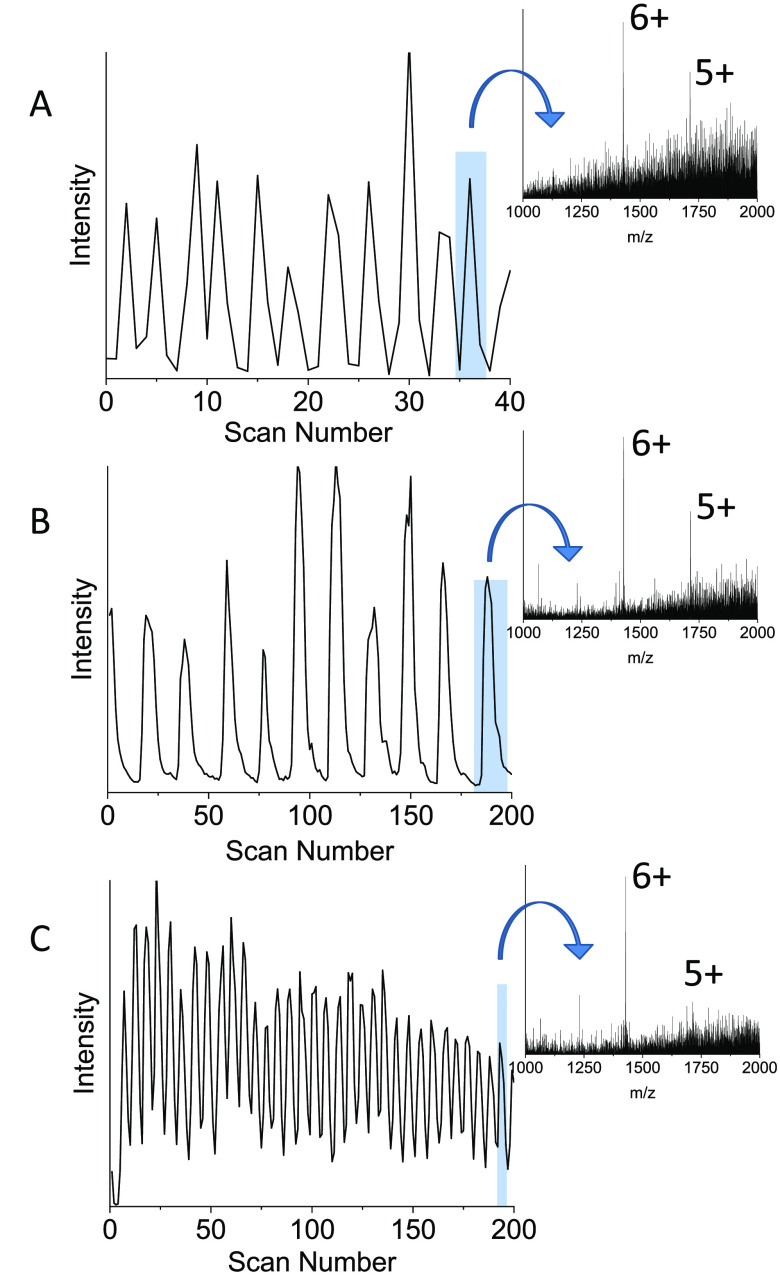
Data acquired using droplets of 60 μM ubiquitin and different acquisition modes on the SYNAPT at varying microdroplet infusion rates. Each mode is accompanied by a mass spectrum extracted from one droplet. (A) TIC obtained from a microdroplet infusion rate of 11 Hz acquired in standard MS mode with a scan time of 0.016 s (1s, ∼40 scans shown). (B) TIC obtained at the same infusion rate but acquired using the fast-scanning acquisition mode (1 s, 200 scans shown). (C) TIC obtained using the fast-scanning mode but at an increased infusion rate of 33 Hz (1 s, 200 scans shown).

The interface utilized between the chip and the mass spectrometer was akin to that presented in Figure 1B with identical channel dimensions employed. Initially, the microdroplet infusion rate generated from a solution of ubiquitin (∼60 μM) reached 11 Hz prior to activation of the fast scanning acquisition mode to confirm droplet detection at the upmost Q-TOF scan rate with detection at this rate observed in Figure 5A. However, the limited number of points gathered per droplet peak results in trilateral peak shapes and does not allow for a further increase in droplet infusion. Activating the fast scanning acquisition and applying a scan time of 1 s allows droplets to be visualized in the drift time real-time display (not shown) as individual peaks similar to that seen within the total ion chromatogram. This real-time display also allowed for further tuning of the instrumentation to improve the stability of infusion and droplet peak shape. Direct visualization of the data acquired in this mode was possible via DriftScope (version 2.8, Waters Corp., Milford, MA, USA). However, for convenience and compatibility with existing software, a script was written and used to unpack the mobility file structure into a continuous “chromatogram-like” output prior to data analysis (Figure 5B and 5C). This total ion chromatogram can be extracted to obtain a mass spectrum for each individual droplet with MassLynx (version 4.2, SCN893, Waters Corp., Milford, MA, USA). Comparing Figure 5A with 5B, each chromatogram has been obtained with a droplet infusion frequency of ∼11 Hz, and Figure 5B has an increased number of mass spectra across each peak. Droplet peaks are therefore sampled at a higher frequency, more accurately representing the underlying peak shape. Increasing the acquisition frequency (i.e., scan rate) allows for a further increase in droplet infusion rate. This is illustrated in Figure 5C, where now the rate is increased to 33 Hz. Further increases in throughput may be possible through optimization of device design, specifically, the channel and stainless-steel emitter dimensions. The microdroplet throughput reported here demonstrates a greater than 10-fold improvement on the detected infusion rate reported by Smith et al. in 2013 for microdroplet reinjection (2.6 Hz).^24^ Operation at an infusion rate of 33 Hz would facilitate the analysis of over 2.8 million samples (Table 1) in one 24 h period. Label-free MS sample throughputs at these speeds would revolutionize screening approaches in areas which rely on indirect measurements or those which require additional labeling procedures due to the MS ability to distinguish compounds by molecular weight. More specifically, applications within synthetic biology and biotechnology have the potential to benefit most from the fusion of high-throughput droplet microfluidics with MS; screening for both improved genetic variations and reaction conditions often requires considerable time and resources. In addition, the high flexibility of microfluidic chip design and the ability to encapsulate cells within droplets also complements the evaluation and miniaturization of synthetic biology assays.

**Table 1:** Sample Throughput Per Unit Time When Continuously Infusing Droplets under Fast Scanning Acquisition Conditions on the Waters SYNAPT Q-TOF Instrument

infusion rate (Hz)	samples/min	samples/h	samples/day (24 h)
11	660	39 600	950 400
33	1980	118 800	2 851 200

### Instrument Comparison

Each instrument platform has advantages and disadvantages in terms of the ease with which the chip-based inlet can be incorporated into the mass spectrometer (Table 2). We found that the Waters nESI source readily coupled to our chip-based inlet. The emitter can be simply inserted through the microsprayer with the bulk of the chip remaining on the *XYZ* stage platform, allowing for both easy access to the fluidic inlets and convenient alteration of the *XYZ* stage location. The Thermo nESI source utilizes a similar facile insertion of the emitter; however, there is no extended platform for the device to be mounted upon. A temporary support was installed (Figure S6) to address this issue; a more robust solution would allow *xyz* adjustment. The most cumbersome of the three arrangements, during both assembly and use, was the Agilent Nanospray source due to the encapsulation of the chip and emitter inside the nESI probe. Insertion of the probe into the source region without due care risked emitter damage, and positioning it in an optimal location between the source inlet and the counter electrode was nontrivial due to the nature of the stage controls. Future modifications would seek to locate the infusion pumps proximal to a modified probe to optimize access to the fluidic connections.

**Table 2:** Table Summarizing the User-Accessible MS Acquisition Scan Speeds, Advantages, and Disadvantages of the Three Instrumental Configurations Assessed in This Article When Coupled with Droplet Microfluidics

instrument type	DTIMS Q-TOF	TWIMS Q-TOF	Orbitrap (FT-MS)
instrument model	Agilent 6560 IM-Q-TOF	Waters SYNAPT G2Si	Thermo Fisher Q Exactive
fastest scan speed	50 scans/s	38 scans/s, 7700 scans/s^a^	30 scans/s
coupling ease	difficult	easy	easy
advantages	grounded emitter	easy coupling	easy coupling
	fastest user accessible scan/s	SONAR technology addition	
disadvantages	stage controls not intuitive	voltage applied to emitter	voltage applied to emitter
	interscan delay not variable	interscan delay	interscan delay not visible
	mounted chip not visible during usage	ESI source accessibility	isotopic resolution lost when increasing scan speed
droplet frequency detected^b^	5 Hz	11 Hz, 33 Hz^a^	6 Hz
droplet size^b^	2.1 nL	0.8 nL, 1.4 nL^a^	0.8 nL

a User-accessible MS acquisition scan speed, droplet frequency, and size detected when SONAR technology is employed.

b Droplet sizes and frequencies stated correspond to the conditions described in this article.

Despite the challenges involved in mounting a chip-based inlet into the Agilent source, the ESI configuration wherein the capillary/emitter is grounded with respect to a source held at lower potential was advantageous to droplet stability. The droplets remained intact and were not prone to coalescence. Application of a positive potential to the emitter, as implemented in the Waters SYNAPT and Thermo Scientific Q Exactive ESI sources, is acceptable for microdroplet generation; however, we had greater difficulties in a droplet reinjection workflow (such as that described by Smith et al.).^23^ Application of a voltage to a pregenerated solution of droplets was found to cause coalescence of the collected droplets.

When considering high-speed acquisition, the Agilent 6560 has the highest user-accessible rate for data collection (50 scans/s); the Waters SYNAPT is similar (∼38 scans/s). The requirement to include some form of delay in which data is not recorded between each acquisition block may cause droplet information to be missed when infusing at such high rates. The Q Exactive FT-MS offers the lowest acquisition speed of the three instruments, and a decrease in mass resolution accompanies operation at the highest acquisition rate ∼30 scans/s. This may curtail uHTS utilization on FT-MS instruments, although the Q Exactive performs well at infusion rates of 1 Hz or lower, which will be adequate for many applications. TOF instrumentation offers increased MS acquisition speeds with the potential to exploit the intrinsically high TOF pusher rate, governed by the acceleration voltage and the longest time-of-flight of a given ion. Currently, the restrictions on this acquisition rate are a consequence of a combination of hardware, system bandwidth and operating system speed, including manufacturers’ software, and practical data file size constraints. While collecting each TOF spectrum individually is conceptually possible without compromising mass-resolving power, one must also consider the effect on the resulting in-spectra dynamic range.

## Conclusions and Outlook

We have demonstrated the coupling of microdroplet microfluidics with mass spectrometry on three instrument platforms from different MS vendors. The microfluidic device with the incorporated emitter is readily interfaced with commercially available nESI sources without extensive modifications, allowing for an infusion of microdroplets up to a rate of 9 Hz. Discrete droplets are easily visualized within the total and extracted ion chromatograms from which mass spectra for each individual droplet can be obtained. Upon assessment of the three instruments, we found the Waters nESI source to be marginally the most accessible due to the ease at which the device could be integrated into the microsprayer adaption, although all sources required some modification and more would be required for optimal permanent use. Application of the voltage directly to the ESI emitter is adequate for infusion but causes droplet coalescence when working with predefined droplets (i.e., droplet reinjection). We are currently working on further modifications to the chips and the sources to prevent this.

All three mass spectrometers utilized in this study were capable of detecting droplets infused at a rate of 5 Hz and above. The Agilent 6560 IM-Q-TOF harnesses the highest speed of 50 scans/s in its commercial configuration, however with additional fast scanning acquisition software available for Waters instruments, detection of increased droplet infusion and has been demonstrated here up to and over a rate of 30 Hz. We believe this can be improved upon further through alteration of the microfluidic channel dimensions and emitter specifications and envision infusion at a rate of 100 Hz achievable in the future. We have demonstrated the ability to infuse droplets of complex salty samples containing small molecules, peptides, and proteins, since we aim to develop a biotechnological application for uHTS, but we envisage a broader class of molecules and accompanying scientific challenges that could benefit from such rapid information-rich analysis.

## Supporting Information Available

Additional mass spectra, instrument assembly photographs, and chip fabrication procedures (PDF)
