# Supplementary material for: Coupling Droplet Microfluidics with Mass Spectrometry for Ultrahigh-Throughput Analysis of Complex Mixtures up to and above 30 Hz
Source: Anal Chem. 2020 Jul 30;92(18):12605–12. doi: 10.1021/acs.analchem.0c02632 (PMC8009470; doi:10.1021/acs.analchem.0c02632)
Supplement: Supplementary file 1 — ac0c02632_si_001.pdf [file ac0c02632_si_001.pdf]

Supporting Information to accompany manuscript - Coupling droplet microfluidics with mass spectrometry for ultra-high-throughput analysis of complex mixtures up to and above 30Hz.

Emily E. Kempa, † Clive A. Smith, ‡ Xin Li, ‡ Bruno Bellina, † Keith Richardson, ¶ Steven Pringle ¶ James Galman, § Nicholas J. Turner § and Perdita E. Barran. †,*

† Michael Barber Centre for Collaborative Mass Spectrometry, Manchester Institute of Biotechnology, Manchester, M1 7DN, UK

‡ Sphere Fluidics Limited, McClintock Building, Suite 7, Granta Park, Great Abington, Cambridge CB21 6GP, UK

¶ Waters Corporation, Stamford Avenue, Altrincham Road, Wilmslow, SK9 4AX, UK

§ Manchester Institute of Biotechnology, Manchester, M1 7DN, UK

1.0 - Device Fabrication 2

2.0 – Instrument Coupling 5

3.0 – Sensitivity Analysis 10

4.0 – Expansion of sample scope 11

1.0 - Device Fabrication

Microfluidic chip designs were drawn using DraftSight software (Dassault Systèmes, Vélizy-Villacoublay, France) before being converted to a film photomask (Micro Lithography Services Ltd, Essex, UK). Device masters were produced by spinning Su-8 2025 epoxy negative photoresist (MicroChem, MA, USA) to a depth of 70 µm upon a silicon wafer. The resulting wafer was baked prior to exposure of the Su-8 2025 using an MJB-4 mask aligner (SUSS MicroTec SE, Garching, Germany), and subsequently, a post-exposure bake was also performed. Designs were developed through the submersion of the exposed wafer in MICRODEPOSIT ™EC solvent (Dow Chemical Company, MI, USA), rinsed, dried and baked again to cement the Su-8 design on to the wafer. The master was placed in a petri dish and a mixture of Sylgard 184 silicone elastomer (10:1, base: curing agent, Dow Chemical Company, MI, USA) added to the dish to the required device thickness. The elastomer was then degassed in a vacuum desiccator, and baked overnight at 70 °C. Removal of the elastomer from the silicon-Su-8 master was performed using a scalpel to expose the design face, and fluidic connections were added using a 1.00 mm biopsy punch (Kai Medical, Solingen, Germany). Microfluidic chip MS designs consist of both a bottom and a top piece, each containing differing design features. The non-feature containing faces of the bottom piece of each design, along with a glass microscope slide, were exposed to oxygen plasma (Harrick Plasma, NY, USA), and the plasma exposed surfaces bound together. A further round of oxygen plasma activation was then performed upon the feature containing surfaces of both the bottom and the top piece of the PDMS device before these two pieces were bound together employing methanol (~10 µL) as a lubricant to ensure the design on each surface were aligned with one another. The device was then baked overnight at 110 °C, prior to insertion of the stainless steel capillary (OD 176 µm, ID 76 µm, Vita Needle Company, Needham, MA, US). Insertion of the capillary was performed under a microscope, ensuring the capillary came into contact with the end of the pre-designed channel, and Elastosil E43 silicone sealant (Wacker Chemie AG, München, Germany) added to the PDMS-capillary interface and allowed to solidify.

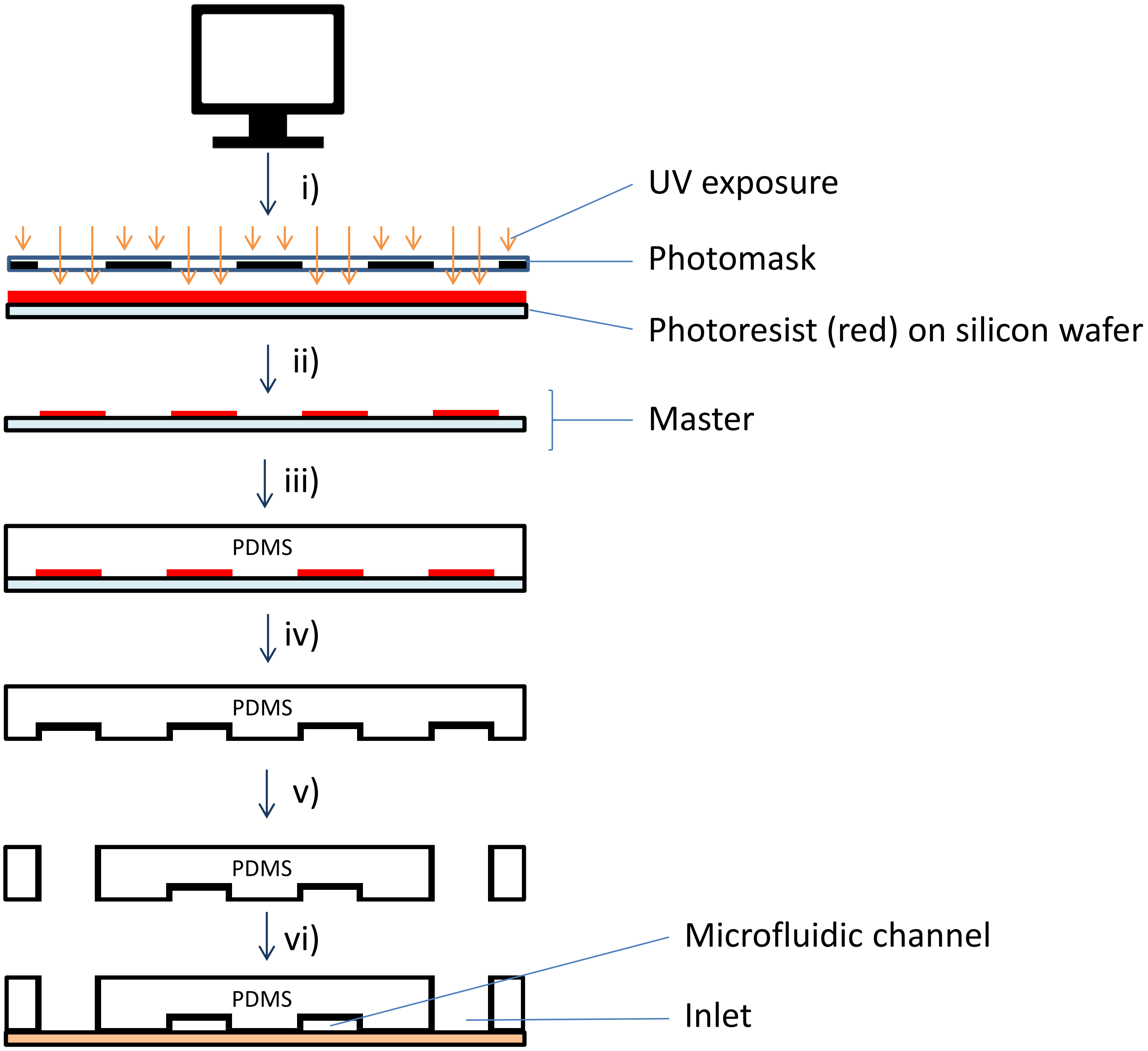

Figure S1: Soft lithography schematic illustrating the steps involved to fabricate a microfluidic chip. i) CAD production of a photomask. ii) Generation of the master by photolithography. iii) Addition of liquid PDMS and curing agent into master. iv) Removal of PDMS from the master. v) Introduction of ports by punching. vi) Binding of PDMS to a glass slide (orange) to create channels.

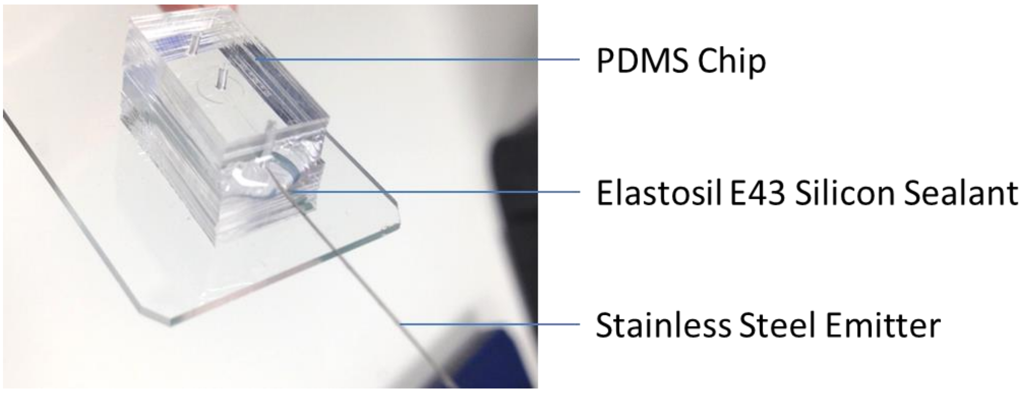

Figure S2: Photograph of the microfluidic-MS chip, indicating the location of Elastosil E43 sealant around the stainless steel emitter.

2.0 – Instrument Coupling

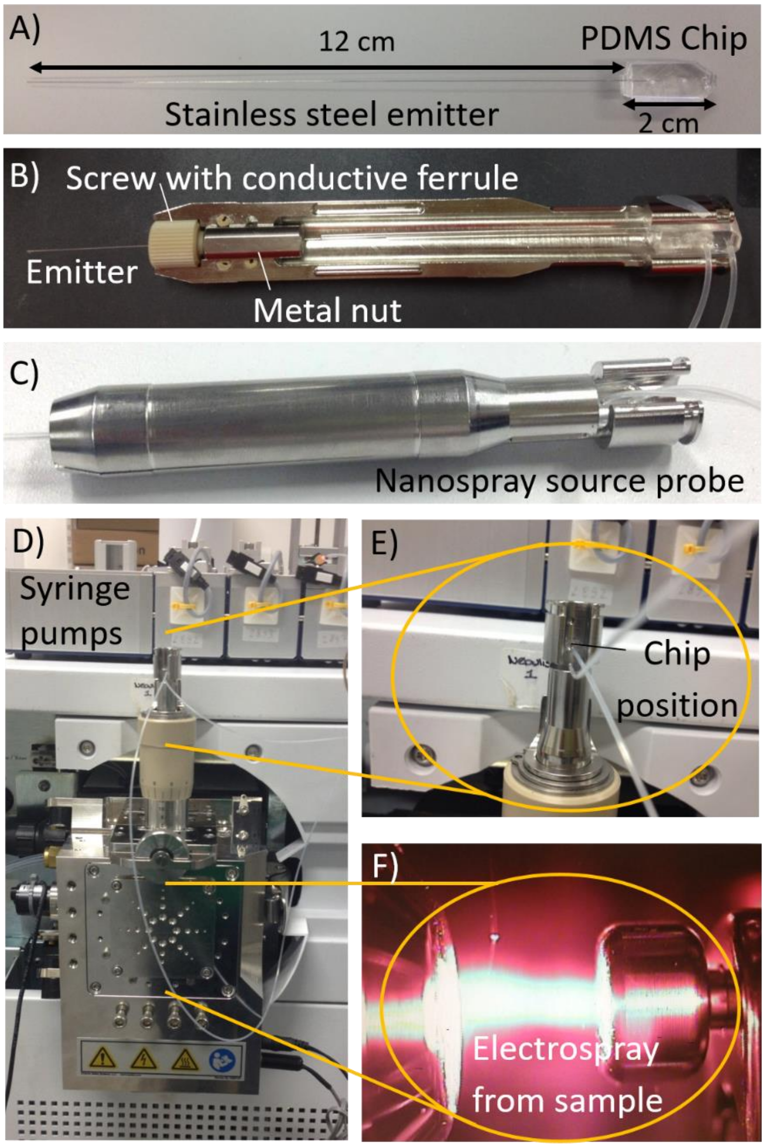

Figure S3: Photographs of Agilent 6560 IM Q-TOF Chip-MS coupling. A) Generation-MS droplet microfluidic chip cut to holder size. B) The bottom section of the nanospray ion source holder indicating chip positioning and added grounding components. C) Enclosed nanoelectrospray source holder ready for insertion into the source. D) Photograph of the Agilent 6560 nanospray ion source indicating chip position and syringe pumps. E) Close up photograph of the droplet microfluidic chip sitting vertically within the holder that inserts into the nanospray ion source. F) Internal camera image of the inside of the nanospray ion source indicating the emitter position between the MS inlet and counter electrode.

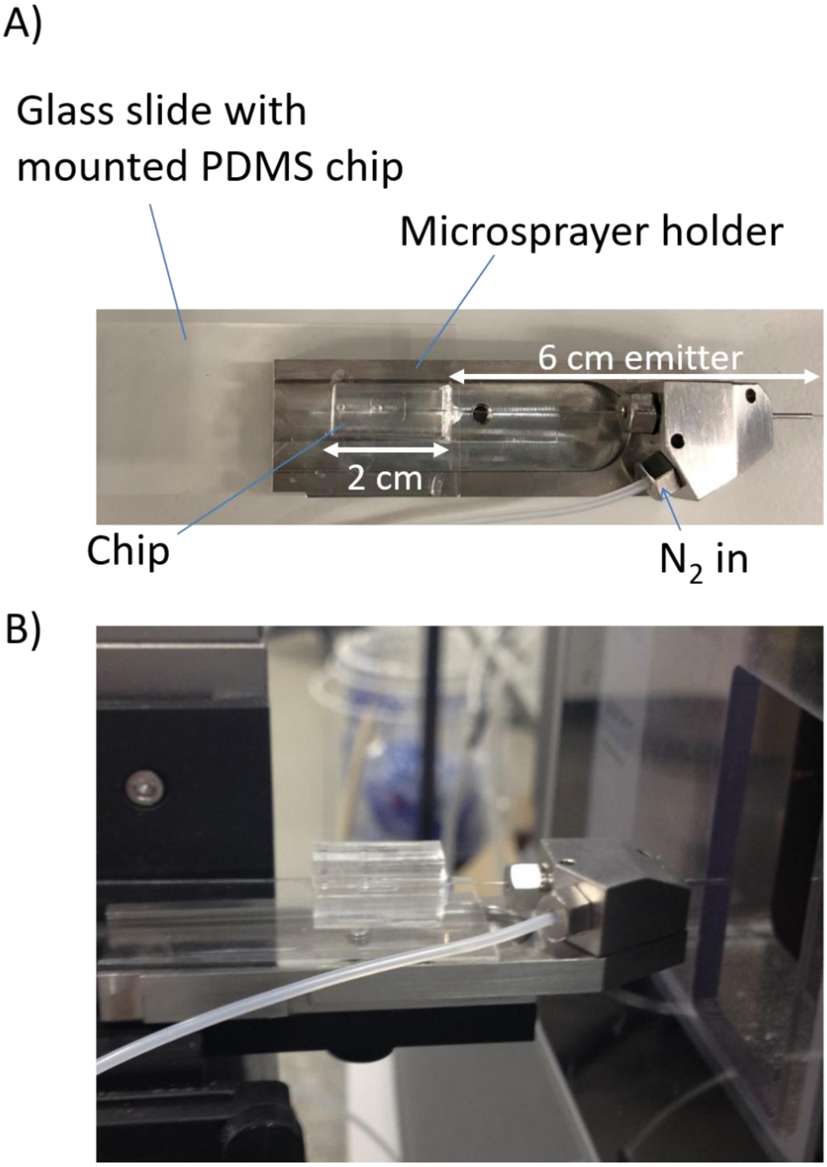

Figure S4: Photographs of Waters Synapt G2si Chip-MS coupling. A) Photographic representation of the microfluidic chip incorporated into the microsprayer device. B) Side on view of the microsprayer adaption when interfaced with the droplet microfluidic chip and mounted on to the nanoelectrospray source.

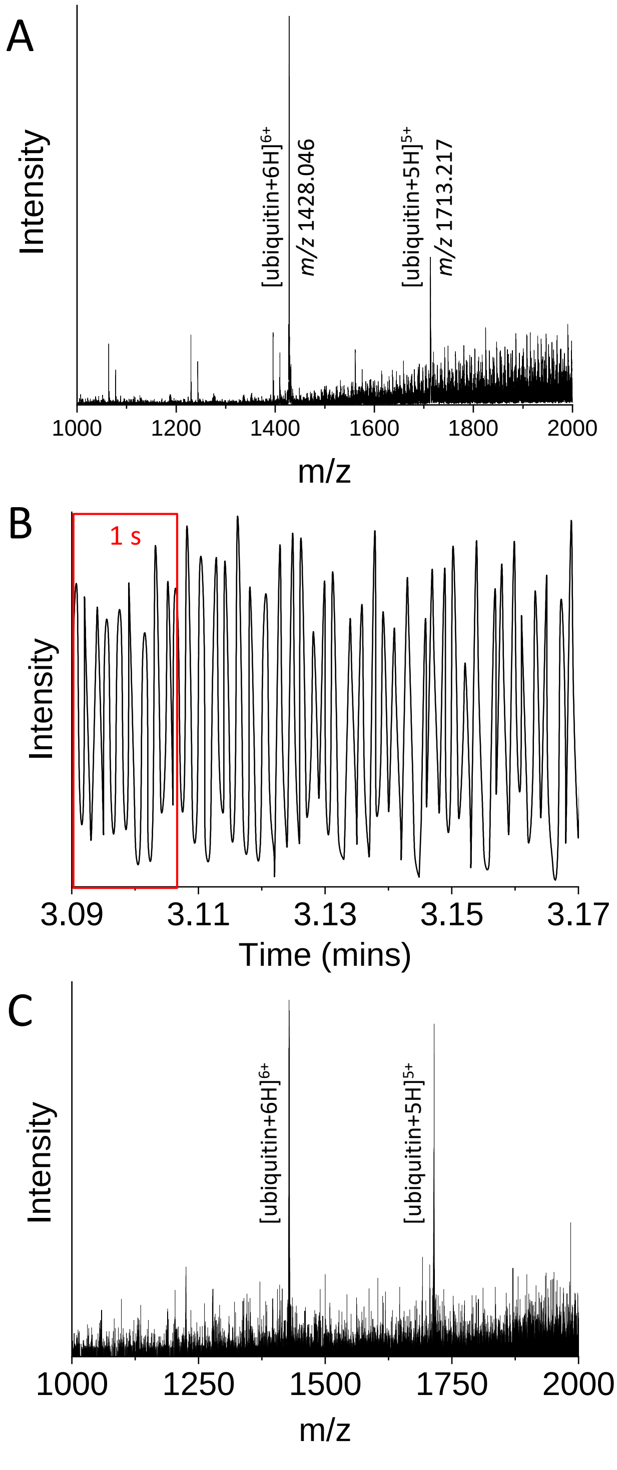

Figure S5: A) nESI mass spectrum of ubiquitin acquired using an SYNAPT G2-si Q ToF mass spectrometer. A 60 µM solution of ubiquitin dissolved in 100 mM ammonium acetate solution was sprayed from a microfluidic chip. B) Total Ion Chromatogram (TIC) acquired during infusion of droplets (~0.8 nL) containing ubiquitin (~100 µM solution) at an infusion rate of approximately 9 droplets per second (Hz). Each individual peak indicates one droplet reaching the SYNAPT G2Si detector. C) Mass spectrum extracted from 1 droplet peak containing ubiquitin, indicating the two major charge states observed under these conditions (1714.59 m/z, 5+ and 1428.43 m/z, 6+).

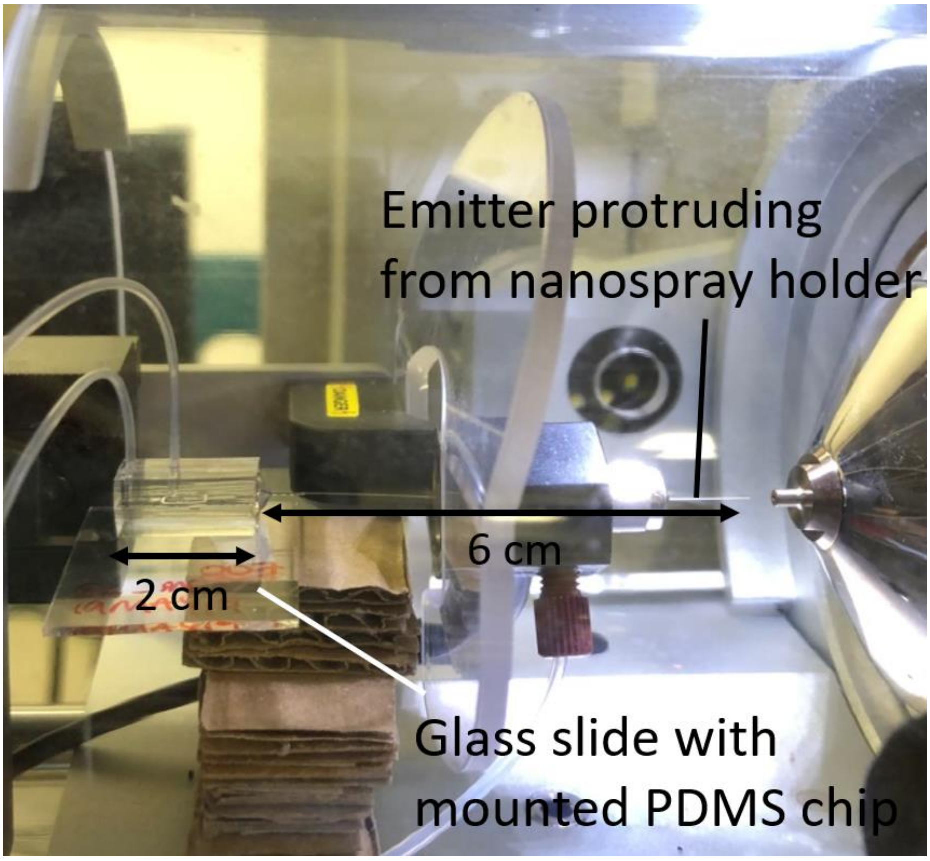

Figure S6: Photograph of a Thermo Fisher Q Exactive Chip-MS coupling, indicating the microfluidic chip position and incorporated emitter.

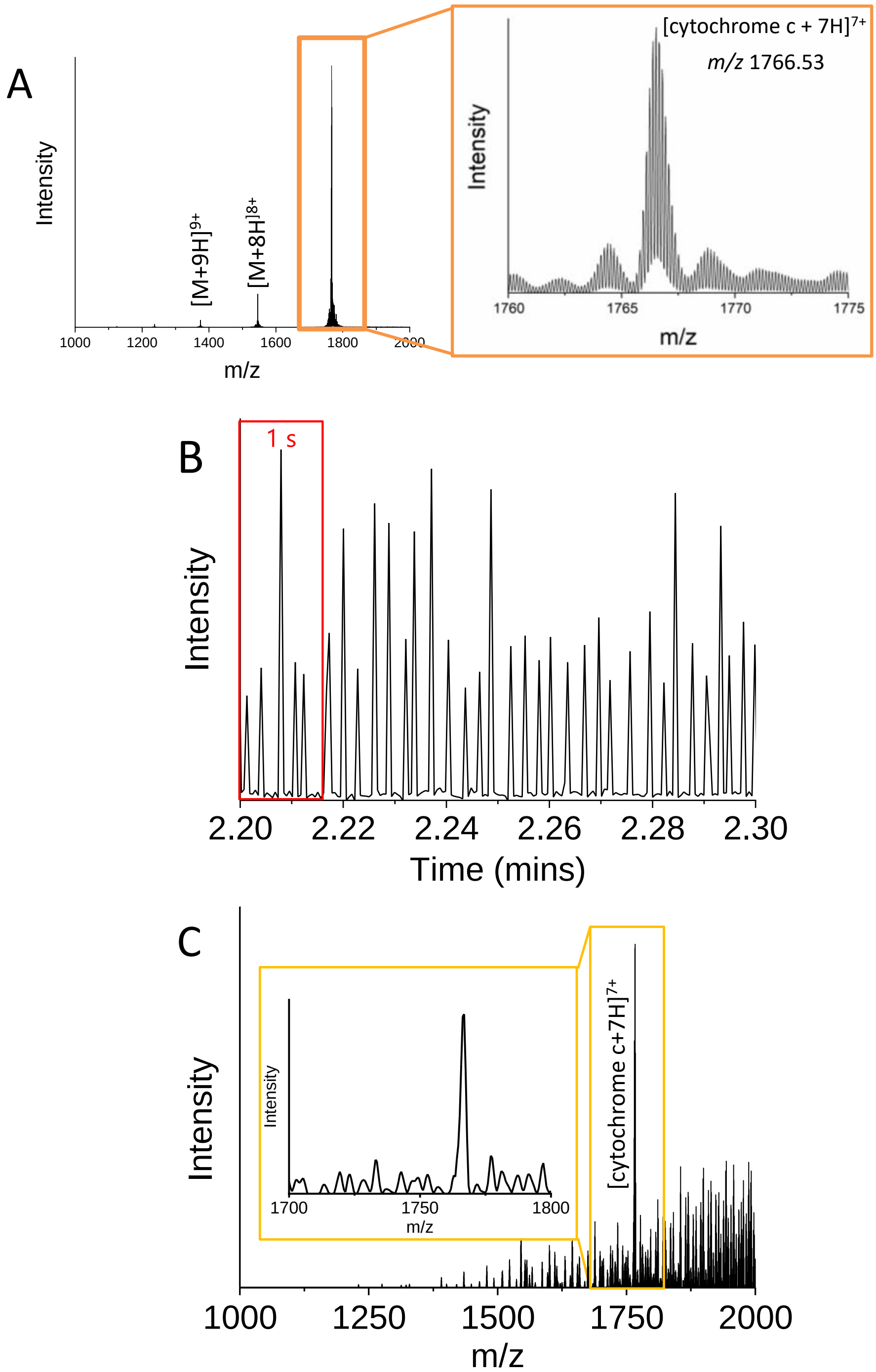

Figure S7: A) nESI mass spectrum of cytochrome c acquired using an Q Exactive FT-MS. A 100 µM solution of ubiquitin dissolved in 100 mM ammonium acetate solution was sprayed from a microfluidic chip using a flow rate of 100 µL/hr and ~2.4 kV capillary voltage. B) Extracted Ion Chromatogram (EIC) acquired during infusion of droplets (~0.8 nL) containing cytochrome c (~100 µM solution) at an infusion rate of approximately 6 droplets per second (Hz). Each individual peak indicates one droplet reaching the Thermo Scientific Q Exactive detector. C) Mass Spectrum (m/z range 1000-2000) acquired from one droplet containing cytochrome c, indicating the most prominent charge state (1766.60 m/z, 7+) in this m/z range. The spectrum inset illustrates the lack of isotopic resolution achieved for the 7+ charge state in comparison to that seen in A.

3.0 – Sensitivity Analysis

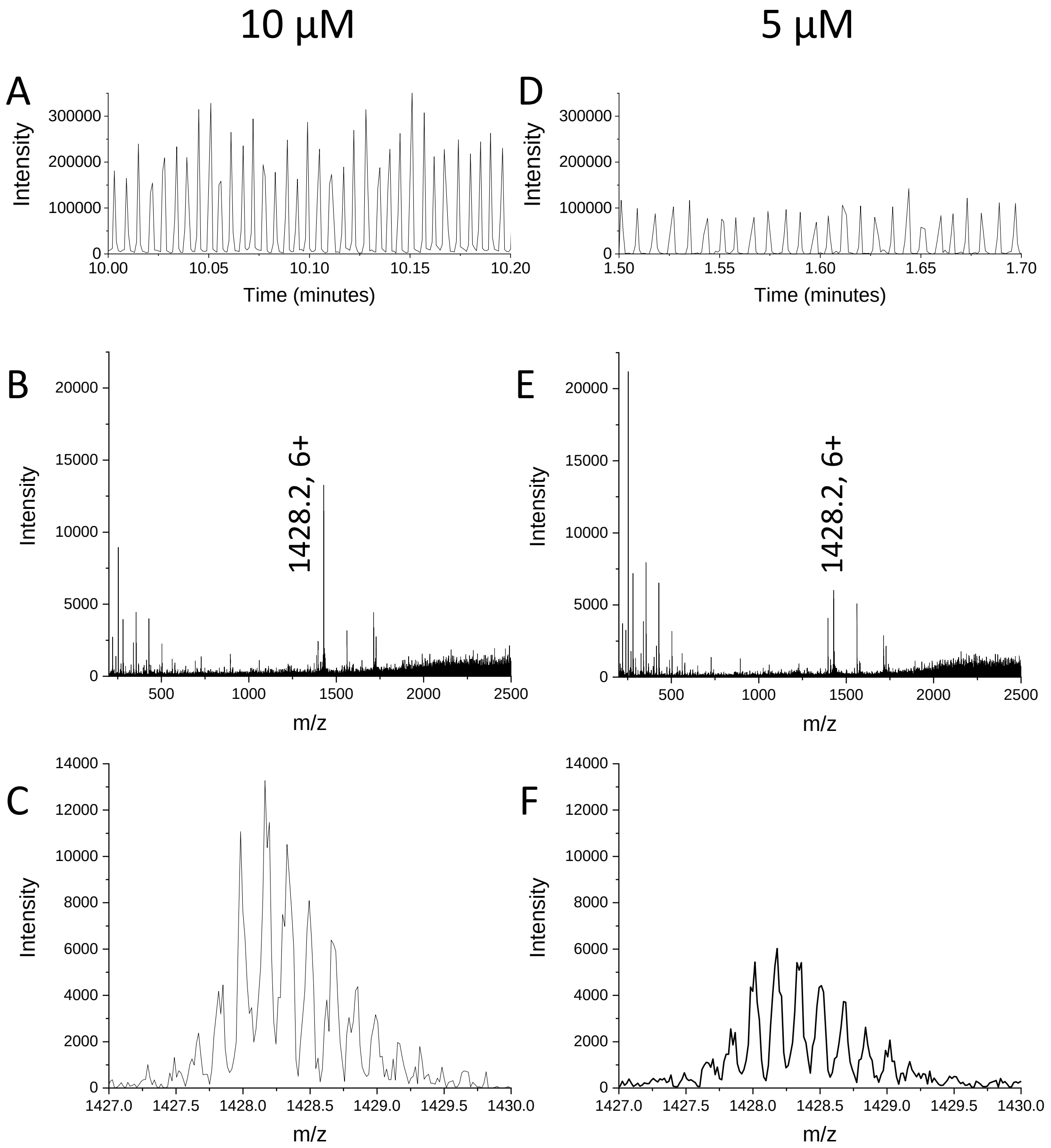

Figure S8: A) Extracted ion chromatogram (m/z 1428) of infused droplets containing 10 µM ubiquitin dissolved in 100 mM ammonium acetate solution. B) Mass spectrum obtained from 1 droplet containing 10 µM ubiquitin solution. C) MS expansion of m/z 1428 ion obtained from 1 droplet containing 10 µM ubiquitin solution. D) Extracted ion chromatogram (m/z 1428) of infused droplets containing 5 µM ubiquitin dissolved in 100 mM ammonium acetate solution. E) Mass spectrum obtained from 1 droplet containing 5 µM ubiquitin solution. F) MS expansion of m/z 1428 ion obtained from 1 droplet containing 5 µM ubiquitin solution.

4.0 – Expansion of sample scope

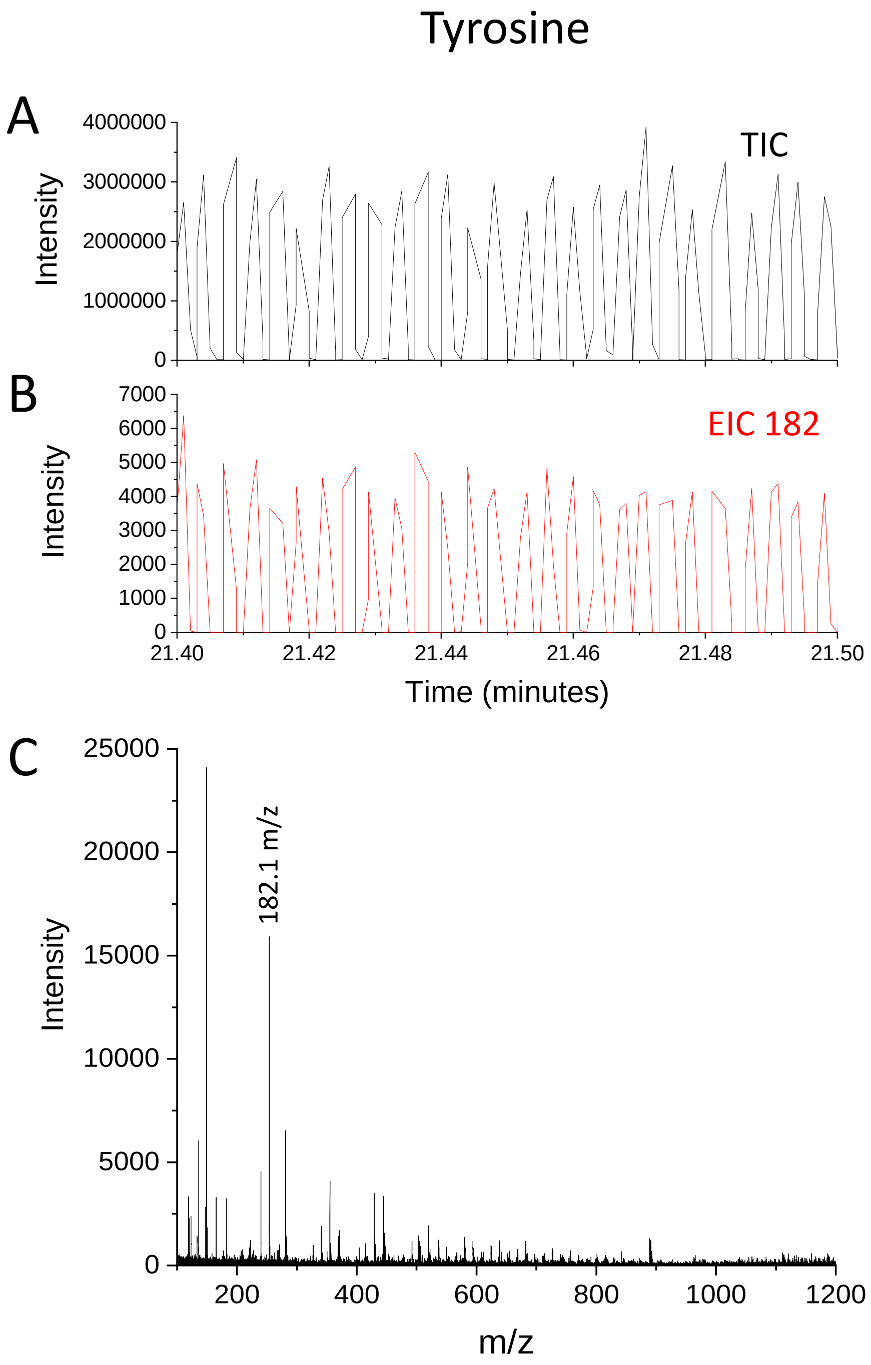

Figure S9: Additional droplet-MS infusion data for the infusion of droplets containing 100 µM L-Tyrosine in aqueous solution containing 0.1% formic acid. Droplet infusion frequency = ~4.5 Hz. A) Total Ion Chromatogram (TIC) of infused Tyrosine droplets. B) Extracted ion chromatogram (EIC) (m/z 182.1) of infused Tyrosine droplets. C) Mass spectrum obtained from 1 droplet peak indicating the major tyrosine analyte ion observed, m/z 182.1, [L-Tyrosine+H]$^+$.

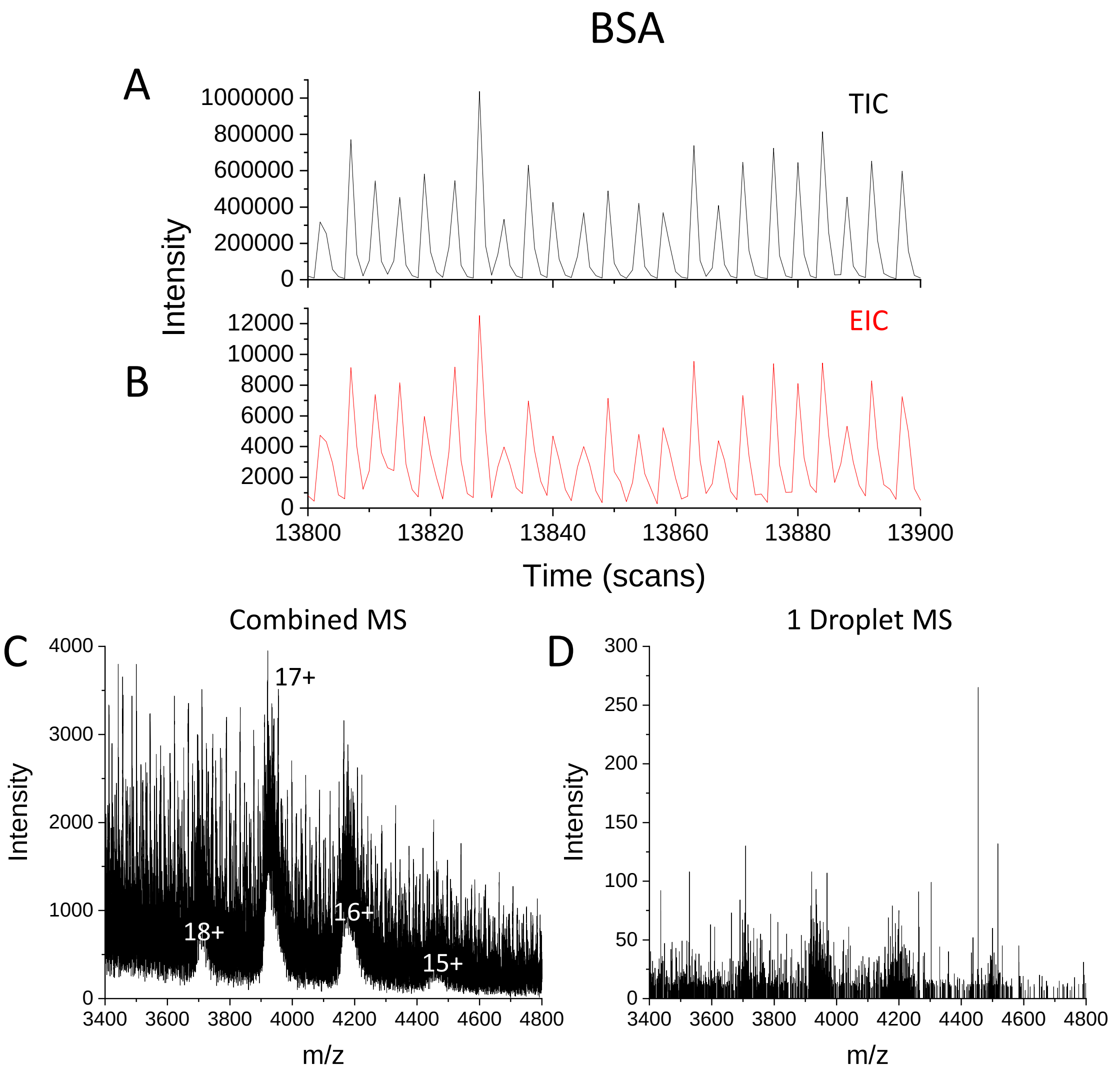

Figure S10: Additional droplet-MS infusion data for the infusion of droplets containing 140 µM Bovine Serum Albumin (BSA, 66 kDa) in aqueous solution containing 100 mM ammonium acetate. For chromatograms, 100 scans (~2.6 s) are shown, with the scan time set to 0.016 s and inter san delay to 0.01 s (total cycle time = 2.6 s). Droplet infusion frequency = ~9 Hz. A) Total Ion Chromatogram (TIC) of infused BSA droplets. B) Extracted ion chromatogram (EIC) (m/z range = 3900-4000) of infused BSA droplets. C) Mass spectrum obtained from combining spectra obtained over ~1 minutes of droplet infusion. The major BSA charge states observed (15+ → 18+) are indicated. D) Mass spectrum obtained from 1 BSA droplet peak.

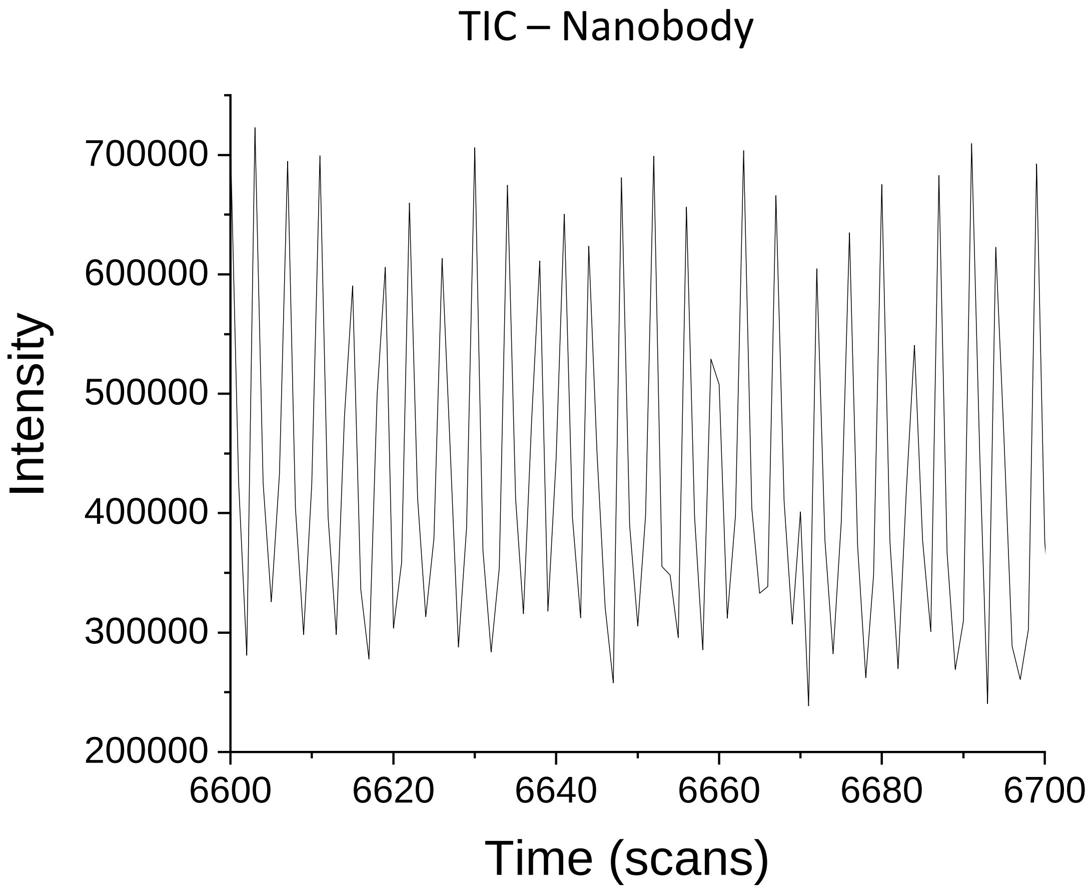

Figure S11: Total ion chromatogram obtained upon the infusion of droplets containing nanobody protein dissolved in a 1 M ammonium acetate solution. 100 scans equivalent to ~ 2.6 s are shown (MS total cycle time = 0.026 s/scan). Droplet infusion frequency = ~ 9 Hz. Droplet size = ~0.8 nL.

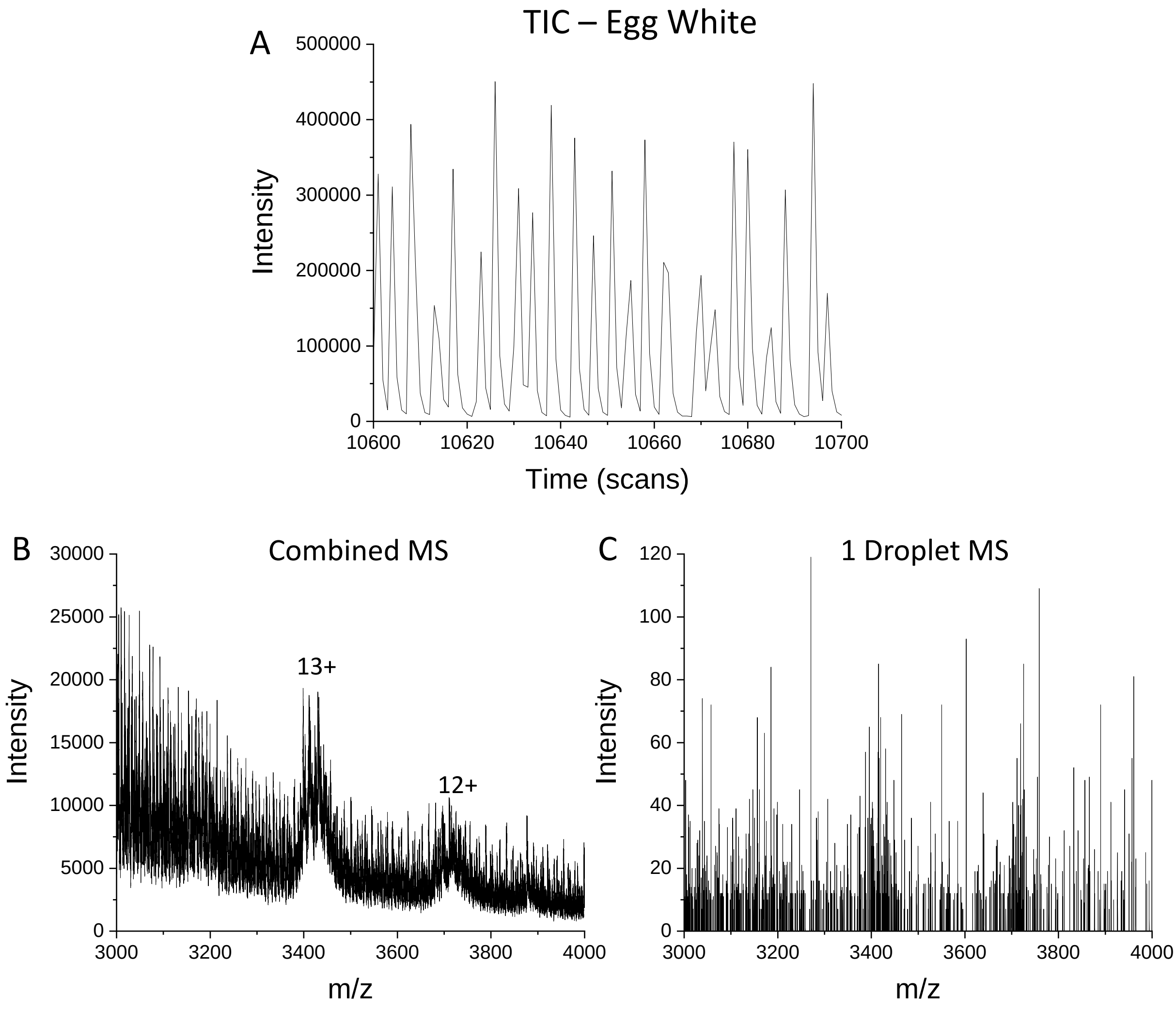

Figure S12: Additional droplet-MS infusion data for the infusion of droplets containing egg white in aqueous ammonium acetate solution (1 M). A) Total ion chromatogram of infused egg white droplets, 100 scans equivalent to ~ 2.6 s are shown (MS total cycle time = 0.026 s/scan). B) Mass spectrum obtained for the infusion of egg white droplets upon combining ~8 minutes of acquisition. Ovalbumin protein (44 kDa) from egg white has been identified in the spectrum with the major charge states of ovalbumin monomer (12+ and 13+) indicated. C) Mass Spectra obtained from 1 egg white droplet.

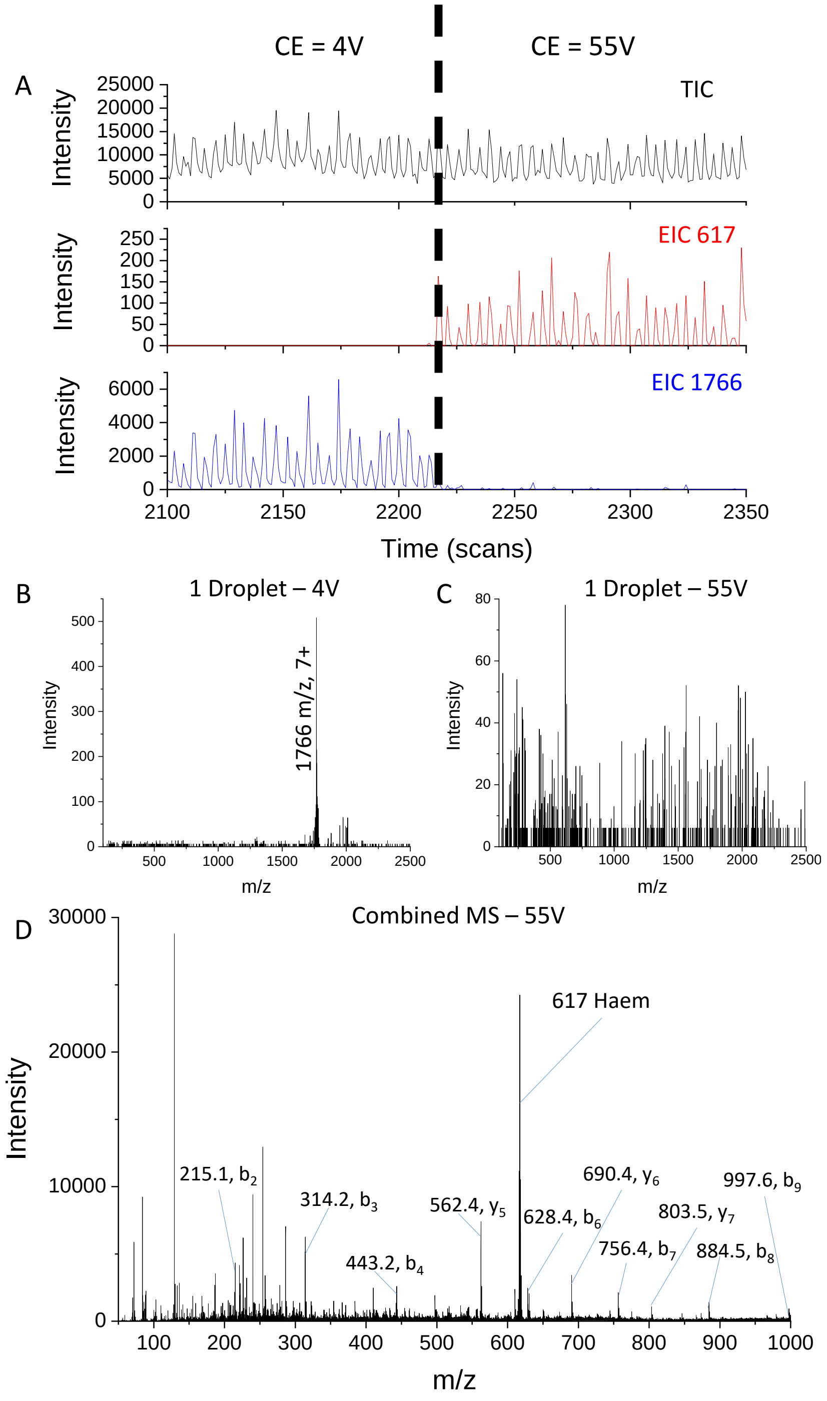

Figure S13: Additional droplet-MS infusion data to indicate the possibility for future MS/MS measurements and top-down fragmentation within droplets. A) Total and extracted ion chromatograms obtained from infusion of cytochrome C droplets. The point at which the collision energy (CE) has been increased from 4V to 55V is indicated by the black dashed line. B) Mass spectrum obtained from 1 droplet when the CE is 4V. Major ion observed is that of [cytochrome C+7]$^{7+}$ due to MS/MS isolation. C) Mass spectrum obtained from 1 droplet when the CE is 55V. A number of ions are observed including Haem (m/z 617) D) Assignment of cytochrome C peptide fragments obtained upon summation of spectra acquired over ~1 minute at CE = 55V.
